# Rare Earth Element Yttrium Modified Mg-Al-Zn Alloy: Microstructure, Degradation Properties and Hardness

**DOI:** 10.3390/ma10050477

**Published:** 2017-04-28

**Authors:** Long Liu, Fulai Yuan, Mingchun Zhao, Chengde Gao, Pei Feng, Youwen Yang, Sheng Yang, Cijun Shuai

**Affiliations:** 1 State Key Laboratory of High Performance Complex Manufacturing, Central South University, Changsha 410083, China; liulong@csu.edu.cn (L.L.); gaochengde@csu.edu.cn (C.G.); fengpei@csu.edu.cn (P.F.); yangyouwen@csu.edu.cn (Y.Y.); 2 Health Management Center, Xiangya Hospital, Central South University, Changsha 410008, China; yuanfulai2017@csu.edu.cn; 3 School of Material Science and Engineering, Central South University, Changsha 410083, China; mczhao@csu.edu.cn; 4 Human Reproduction Center, Shenzhen Hospital of Hongkong University, Shenzhen 518053, China; tobyys2000@aliyun.com; 5 Key Laboratory of Organ Injury, Aging and Regenerative Medicine of Hunan Province, Changsha 410008, China

**Keywords:** AZ61 magnesium alloy, microstructure, degradation properties, hardness

## Abstract

The overly-fast degradation rates of magnesium-based alloys in the biological environment have limited their applications as biodegradable bone implants. In this study, rare earth element yttrium (Y) was introduced into AZ61 magnesium alloy (Mg-6Al-1Zn wt %) to control the degradation rate by laser rapid melting. The results showed that the degradation rate of AZ61 magnesium alloy was slowed down by adding Y. This was attributed to the reduction of Mg_17_Al_12_ phase and the formation of Al_2_Y phase that has a more active potential, which decreased galvanic corrosion resulting from its coupling with the anodic matrix phase. Meanwhile, the hardness increased as Y contents increased due to the uniform distribution of the Al_2_Y and Mg_17_Al_12_ phases. However, as the Y contents increased further, the formation of excessive Al_2_Y phase resulted in the increasing of degradation rate and the decreasing of hardness due to its agglomeration.

## 1. Introduction

Magnesium-based alloys have aroused keen attention as biodegradable bone implants due to their unique biodegradable characteristics, proper mechanical properties and favorable biocompatibility [1,2,3,4,5,6]. The widely used Mg-Al-Zn (AZ series) alloys belong to a magnesium-based alloy, which exhibits high strength and certain degradation resistance [7,8,9,10]. Nevertheless, it still needs to further enhance degradation resistance in order to have biological applications [11,12,13]. The rare earth elements such as neodymium (Nd), gadolinium (Gd) and yttrium (Y) have a beneficial effect in increasing degradation resistance and enhance the mechanical properties of magnesium alloys [14,15,16,17,18].

Many research works have been carried out on magnesium alloys with rare earth elements. Zhang et al. [19] reported that alloying cerium (Ce) could improve mechanical properties and corrosion resistance of cast Mg-4Al-based alloy. Liu et al. [20] reported that the addition of Lanthanum (La) could enhance the corrosion properties of AM60 alloy. Yttrium (Y), a rare earth element, has hexagonal close-packed crystal structure which is the same as that of magnesium, and the atomic radius of Y (0.18 nm) is close to that of magnesium (0.16 nm) [21]. Thus, its solid solubility limit in magnesium alloys can reach up to 11.4 wt % [22]. Moreover, Y has the same standard electrochemical potential with magnesium (−2.372 V) [23,24]. Qi et al. investigated the effects of Y on the microstructure and mechanical properties of as-cast Mg-6Zn-1Mn alloy. The results showed that Y could improve its mechanical properties significantly, and the alloy with Y content of 6.09 wt % has the best mechanical properties [25]. Luo et al. studied the corrosion resistance property and the corrosion evolution of as-cast AZ91 alloy with rare earth Y. They found that the proper amount of Y addition could improve the corrosion resistance of as-cast AZ91 alloys effectively [26].

Laser rapid melting has the characteristic of rapid solidification. The cooling rate during laser melting usually reaches up to 10^5^ K/s, which can inhibit grain growth and refine the grains [27]. Meanwhile, laser rapid melting can reduce the composition segregation. Furthermore, laser rapid melting is a non-equilibrium process which can increase the solid solubility of the alloy elements [28].

In this work, the AZ61 magnesium alloys (Mg-6Al-1Zn wt %) with different Y contents (0, 1, 2, 3, 4 wt %) were prepared using laser rapid melting. The microstructure was studied by optical microscopy (OM), X-ray diffraction (XRD) and scanning electron microscopy (SEM) with energy dispersed spectroscopy (EDS). The degradation properties were analyzed by the immersion experiments. In addition, the hardness was measured by Vickers hardness tests.

## 2. Results and Discussion

### 2.1. Microstructure

**Figure 1:**
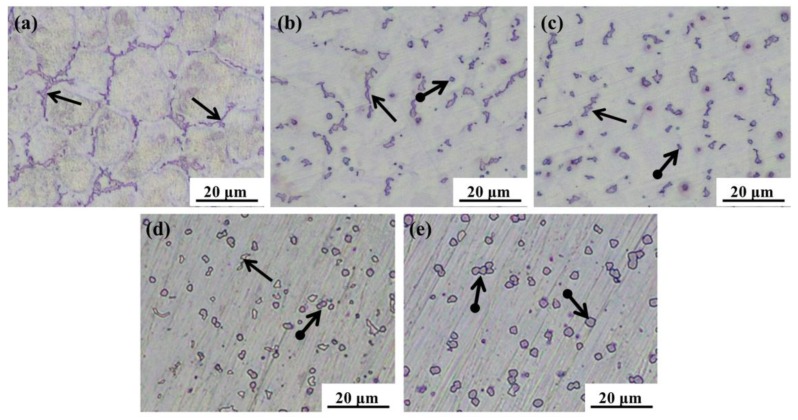
Optical micrographs of AZ61 magnesium alloys with different Y contents: (**a**) 0 wt %; (**b**) 1 wt %; (**c**) 2 wt %; (**d**) 3 wt % and (**e**) 4 wt %.

**Figure 2:**
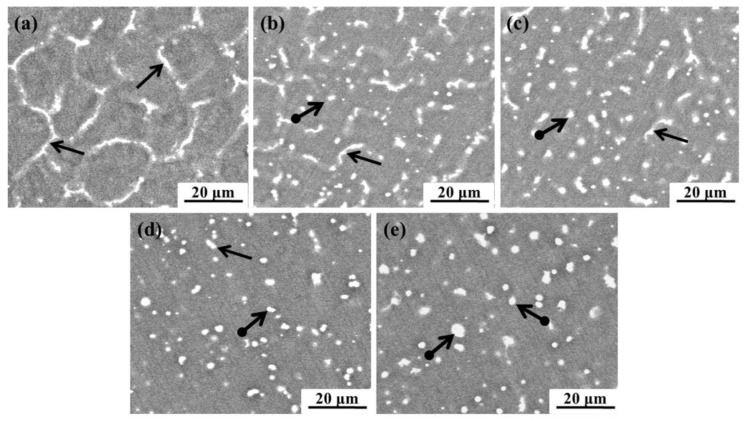
SEM micrographs of AZ61 magnesium alloys with different Y contents: (**a**) 0 wt %; (**b**) 1 wt %; (**c**) 2 wt %; (**d**) 3 wt % and (**e**) 4 wt %.

The microstructures of the AZ61 magnesium alloys with different amounts of Y added were examined by optical microscopy (Figure 1) and scanning electron microscopy (Figure 2) respectively. AZ61 magnesium alloy consisted of the magnesium matrix and network precipitates (pointed by black arrows) which were distributed mainly at grain boundaries (Figure 1a and Figure 2a). After adding Y, the network precipitates decreased and a small amount of granulous precipitates (pointed by black arrows with a round tail) appeared (Figure 1b and Figure 2b). The granulous precipitates increased as the Y contents increased and were distributed uniformly as Y reached 2 wt % (Figure 1c and Figure 2c). However, as the Y contents further increased, the granulous precipitates tended to be predominant and agglomerate (Figure 1d,e and Figure 2d,e).

**Figure 3:**
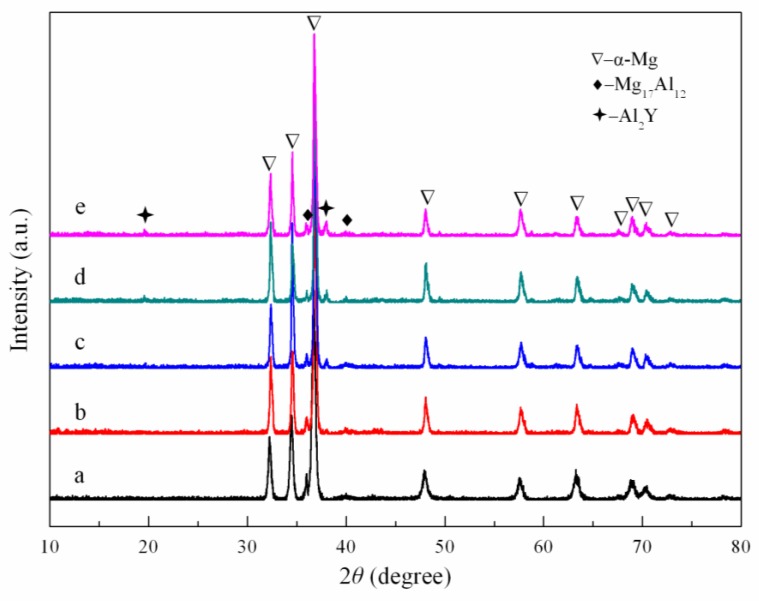
X-ray diffraction (XRD) patterns of AZ61 magnesium alloys with different Y contents: (**a**) 0 wt %; (**b**) 1 wt %; (**c**) 2 wt %; (**d**) 3 wt % and (**e**) 4 wt %.

**Figure 4:**
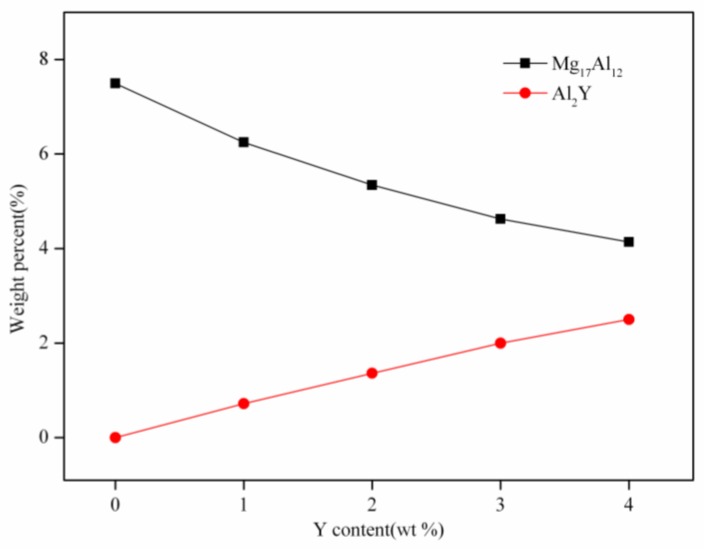
The weight percent of Mg_17_Al_12_ and Al_2_Y phases in AZ61 magnesium alloys with Y addition.

The XRD patterns of the AZ61 magnesium alloys with different Y contents were exhibited in Figure 3. Only the α-Mg and Mg_17_Al_12_ phase were detected in the AZ61 magnesium alloy (Figure 3a). After adding Y, a new Al_2_Y phase was formed and the corresponding peak intensity gradually increased as Y increased. Meanwhile, the peak intensity of the Mg_17_Al_12_ phase decreased. This implied the reduction of the Mg_17_Al_12_ phase and the increase of the Al_2_Y phase. Reference intensity ratio (RIR) method was used to quantify the weight percent of the Mg_17_Al_12_ phase and Al_2_Y phase. A more complete view on the weight percent of the Mg_17_Al_12_ phase and Al_2_Y phase as influenced by Y content was given in Figure 4. It could be observed that the weight percent of the Mg_17_Al_12_ phase decreased from 7.5% in the AZ61 magnesium alloy to 4.1% in the AZ61 magnesium alloy with 4 wt % Y, while that of Al_2_Y increased from 0% in the AZ61 magnesium alloy to 2.5% in the AZ61 magnesium alloy with 4 wt % Y. In general, the tendency of elements to form stable compounds was in positive correlation with the electronegativity difference between elements. The electronegativity values of Y, Mg and Al were 1.22, 1.31 and 1.61 respectively, from which it could be deduced that Y was prone to react with Al to form Al-Y compound [29]. Thus, the Al_2_Y phase increased gradually as Y increased, while the Mg_17_Al_12_ phase decreased.

**Figure 5:**
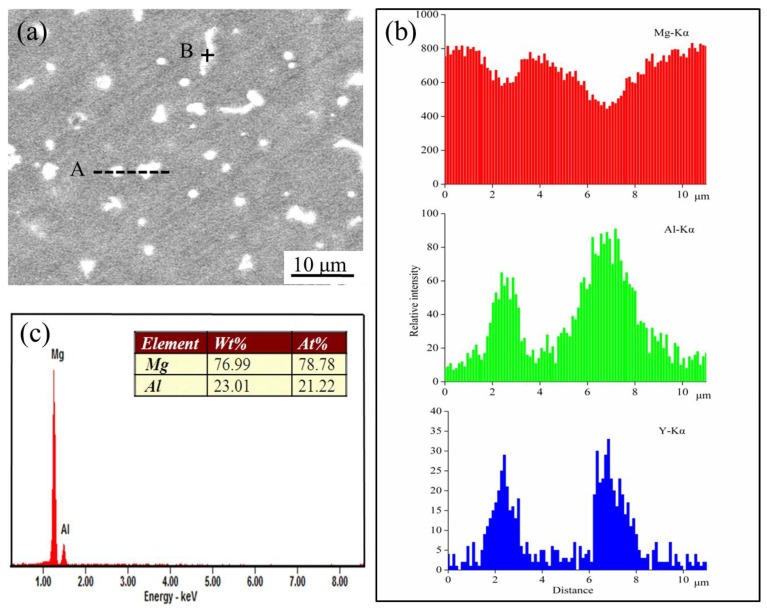
SEM image of (**a**) AZ61 magnesium alloy with 2 wt % Y and energy dispersive spectroscopy (EDS) patterns of (**b**) A line and (**c**) B point in (**a**).

The morphology and compositions of the second phases in AZ61 magnesium alloy with 2 wt % Y were studied by EDS. It was presented that magnesium content reduced while Al and Y increased across the granulous particles (Figure 5b). Thus, it was reasonable to conclude that the granulous particle was in the Al_2_Y phase. Meanwhile, the rod-shaped phase had a Mg/Al ratio (78.78/21.22) (Figure 5c) which was close to that of Mg_17_Al_12_, and was thereby identified as Mg_17_Al_12_ phase.

**Figure 6:**
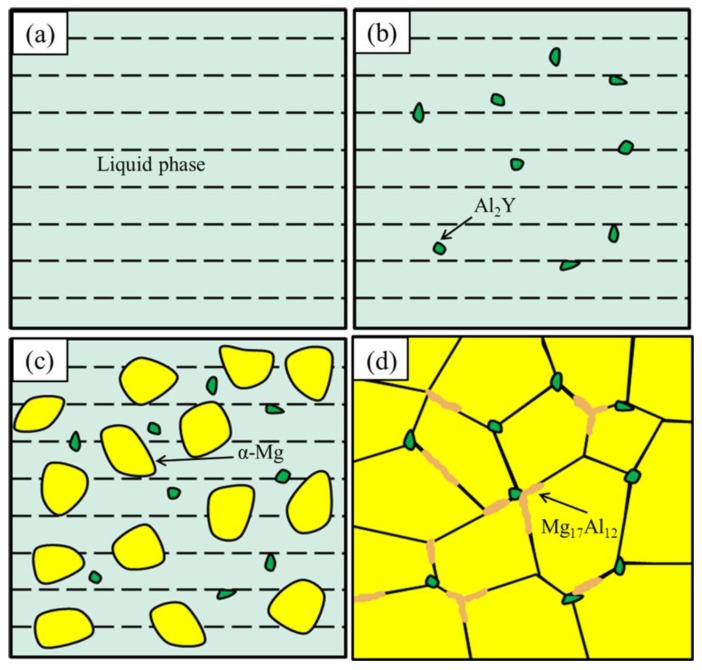
Schematic diagram of phase formation in the AZ61 magnesium alloy with Y (**a**) liquid phase; (**b**) Al_2_Y phase precipitation; (**c**) α-Mg nucleation; (**d**) Mg_17_Al_12_ formation.

The three phases (α-Mg, Al_2_Y and Mg_17_Al_12_) were exactly determined in the AZ61 magnesium alloy with Y. The schematic diagram of the phase formation in the alloy was shown in Figure 6. The first precipitated phase in the solidification of the high-temperature liquid phase was the Al_2_Y phase. This could be explained by the Al_2_Y phase having the highest melting point (1485 °C) among the three phases (Figure 6b) [30]. Afterwards, the α-Mg phase with the melting point of 650 °C started nucleation (Figure 6c). Then, the remained Al atoms precipitated in Mg_17_Al_12_ (437 °C) which distributed on the α-Mg grain boundaries (Figure 6d).

### 2.2. Hardness

**Figure 7:**
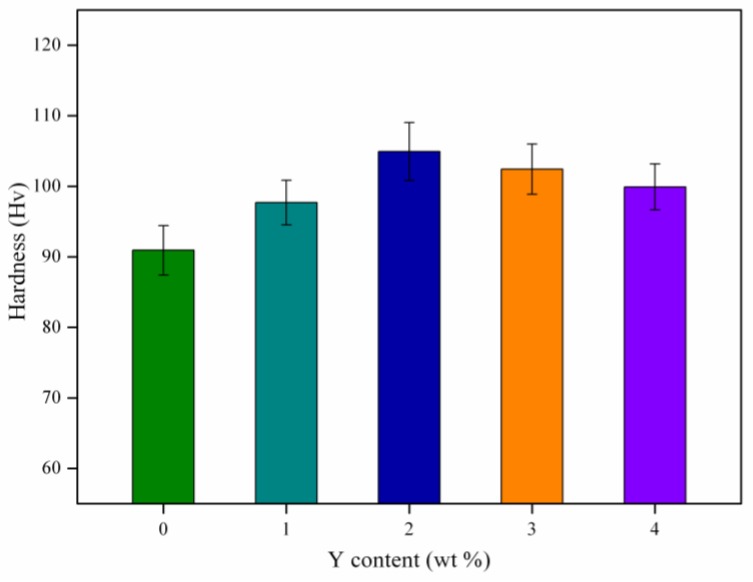
Hardness of AZ61 magnesium alloys with different Y contents.

The hardness of AZ61 magnesium alloys with different Y contents was shown in Figure 7. The hardness of AZ61 magnesium alloy was 90.9 Hv. The hardness continuously increased as Y increased from 0 wt % to 2 wt %. The optimal hardness was 104.9 Hv when the Y content was 2 wt %. However, it decreased as the Y contents further decreased. The increase of hardness was attributed to the uniform distribution of Al_2_Y and Mg_17_Al_12_ phases which acted as a second phase strengthening agent in the alloy matrix. As the Y contents further decreased, the excessive Al_2_Y phase formed in the alloy tended to aggregate. Thus, the structure of the alloy became uneven and the hardness of the alloy decreased.

### 2.3. Degradation Properties

**Figure 8:**
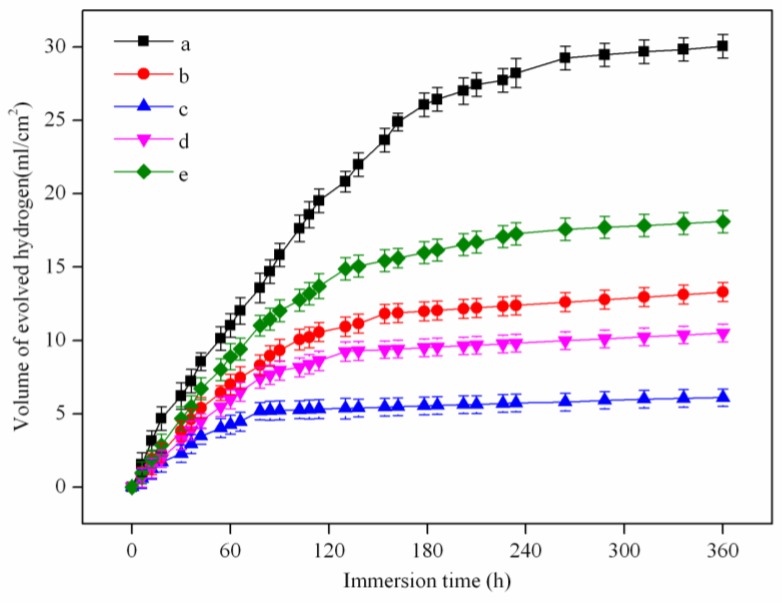
The hydrogen evolution volume of AZ61 magnesium alloys with different Y contents immersed in the simulated body fluid (SBF) for 360 h: (**a**) 0 wt %; (**b**) 1 wt %; (**c**) 2 wt %; (**d**) 3 wt % and (**e**) 4 wt %.

**Figure 9:**
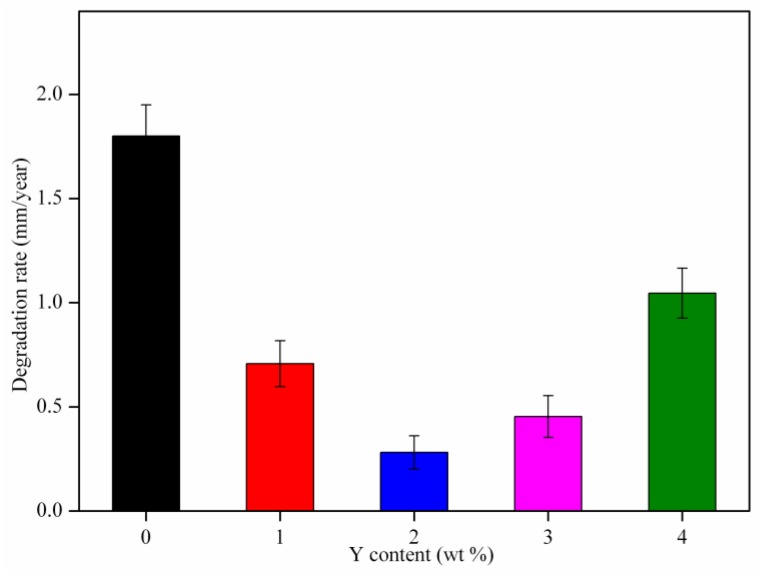
Degradation rates of AZ61 magnesium alloys with different Y contents after immersion in SBF solution for 7 days.

Immersion tests were applied to study the degradation properties of AZ61 magnesium alloys with different Y contents. The hydrogen evolution volume varied with the immersion time, as shown in Figure 8. The hydrogen evolution volume of the alloys increased rapidly in the early stages of immersion and then increased slowly, which indicated a reduction of degradation rate. The hydrogen evolution volume of AZ61 magnesium alloys with Y contents of 0, 1, 2, 3 and 4 wt % were 30.1 mL/cm^2^, 13.3 mL/cm^2^, 6.1 mL/cm^2^, 10.5 mL/cm^2^ and 18.1 mL/cm^2^ after immersion for 360 h, respectively. It was observed that the degradation rate reduced remarkably with adding Y up to 2 wt %, while a further increase of Y resulted in an increased degradation rate. The degradation rates of the AZ61 magnesium alloys with different Y contents were calculated according to mass loss test (Figure 9). The results were consistent with that of hydrogen evolution analysis, which showed that AZ61 magnesium alloy with 2 wt % Y exhibited the lowest degradation rate (0.28 mm/year). The degradation rate of AZ61 magnesium alloy with 2 wt % Y was lower than the reported value of WE43 magnesium alloy (0.85 mm/year) [31].

**Figure 10:**
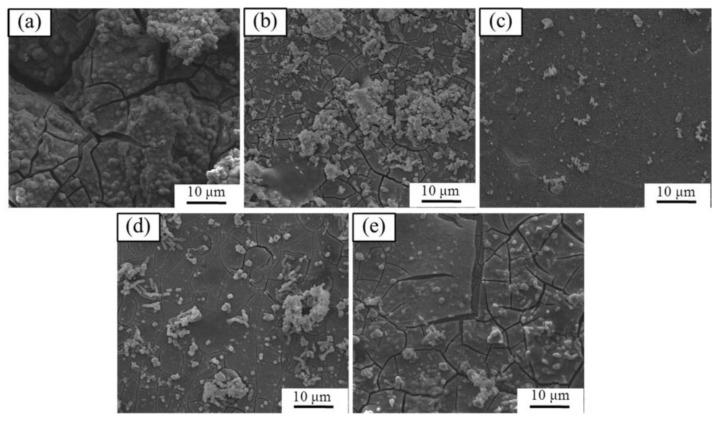
SEM degradation morphology of AZ61 magnesium alloys with different Y contents after immersion for 120 h: (**a**) 0 wt %; (**b**) 1 wt %; (**c**) 2 wt %; (**d**) 3 wt % and (**e**) 4 wt %.

The degradation morphology of the AZ61 magnesium alloys with different Y contents after immersion for 120 h was shown in Figure 10. Obviously, the alloys were covered with a degradation product film which presented some cracks. The appearance of cracks was believed to be caused by the dehydration of the degradation product film after drying in ambient atmosphere. The AZ61 magnesium alloy exhibited a severely corroded surface with many cracks (Figure 10a). After adding 1 wt % Y, the surface of the alloy presented relatively shallow cracks. When the Y contents was 2 wt %, the integrated degradation film without a crack formed, which implied that the degradation degree of the alloy was relatively low (Figure 10c). As the Y contents further increased, the cracks of the degradation product film gradually increased (Figure 10d,e).

It could be concluded that the appropriate addition of rare earth element Y could enhance degradation resistance of the AZ61 magnesium alloy. In general, the Mg_17_Al_12_ phase acted as the cathode with respect to the magnesium matrix, which facilitated the degradation of the AZ61 magnesium alloy [32]. After adding Y, Y reacted with Al to form the Al_2_Y phase, which reduced the amount of Mg_17_Al_12_ phase on the grain boundaries. Furthermore, the Al_2_Y phase has more active potential [33]. Thus, adding Y could suppress the galvanic corrosion of the alloys, which enhanced the degradation resistance. However, as the Y contents further increased, the excessive Al_2_Y phase accelerated galvanic corrosion, resulting in the increase of degradation rate.

## 3. Experimental Procedure

### 3.1. Materials Preparation

The spherical AZ61 magnesium alloy powders were purchased from Tangshan Weihao Materials Co., Ltd. (Tangshan, China, average particle size 70 µm) and irregular-shaped Y powders were obtained from Shanghai Naiou Nano technology Co., Ltd. (Shanghai, China, average particle size 20 µm). The powder mixtures with different Y contents (0, 1, 2, 3 and 4 wt %) were prepared through ball milling in a mixed gas environment (1 vol % SF_6_ and 99 vol % CO_2_). The rotation speed was fixed at 450 rpm (revolution per minute) in the course of ball milling and the milling time was 2 h.

The AZ61 magnesium alloys with different Y contents (0, 1, 2, 3, 4 wt %) were prepared using a homemade laser rapid melting system. It consists of a fiber laser, a focus system, a gas protection device and a computer control system. The fiber laser has a maximum output power of 110 W. The minimum spot diameter of the laser beams is 50 μm. More details of the system are available in the reference [34]. The processing parameters were as follows: laser scanning rate 200 mm/min, laser spot 150 μm and laser power 80 W. The powder mixtures were melted layer by layer in the sealed building chamber protected by argon gas. Then, the samples of alloys (10 mm × 10 mm × 5 mm) were built up.

### 3.2. Materials Characterization

The prepared alloy specimens were ground with abrasive papers grading from 1000 to 2000 grit and mechanically polished on cotton cloth with 0.5 μm diamond paste, followed by being etched for 10 s with the acetic picral solution (10 mL of acetic acid, 70 mL of ethanol (99.8% *v*/*v*), 4.2 g of picric acid, and 10 mL of distilled water), then the metallurgical structure of alloys was studied by an optical microscopy (OM, Olympus BHM, Osaka, Japan). The composition distribution of the alloys was studied using scanning electron microscopy (SEM, QUANTA FEG250, FEI Company, Hillsboro, OR, USA) and energy dispersive spectroscopy (EDS, JSM-5910LV, JEOL, Tokyo, Japan). The phase compositions were analyzed through X-ray diffraction (XRD, D8 Advance, Bruker Inc., Karlsruhe, Germany) using Cu-Kα radiation at 15 mA and 30 kV with scattering angles ranging from 10° to 80°, step size 0.02° and scanning speed 8°/min. X-ray diffraction patterns were identified by comparing the diffraction patterns with the standard ICDD-PDF cards. The quantitative phase analysis was conducted by means of the reference intensity ratio method [35].

Vickers hardness tests were performed by a Vickers microindenter (HXD-1000TM/LCD, Digital Micro Hardness Tester, Shanghai Taiming Optical Instrument Co. Ltd, Shanghai, China) with a load of 2.45 N and loading time of 15 s. Ten indents were made for each sample. The hardness was expressed as a mean and standard deviation of these 10 readings. The dimensions of alloys used for the immersion tests were 10 mm × 10 mm × 5 mm. Immersion tests were operated at 37 ± 0.5 °C in simulated body fluid (SBF) (the ratio of the surface area to solution volume was 1 cm^2^:100 mL). The SBF that has similar ion concentrations to those of human blood plasma was prepared according to the protocol described by Kokubo et al. [36]. In short, the relevant reagent grade chemicals (CaCl_2_, K_2_HPO_4_·3H_2_O, KCl, NaCl, MgCl_2_·6H_2_O, NaHCO_3_ and Na_2_SO_4_) were dissolved in distilled water at the appropriate amounts. The pH of the solution was buffered to physiological pH (pH = 7.4) by adding tri-hydroxymethyl-aminomethane and hydrochloric acid. The hydrogen evolution volume was monitored during the immersion. After immersion for 7 days, each sample was removed from the solution and washed with distilled water. A chromic acid solution (200 g/L Cr_2_O_3_ + 10 g/L AgNO_3_) was used to remove the degradation products on the sample surface before mass loss measurement. Five samples were measured for each group to obtain reproducible results. The degradation rates (mm/year) were calculated according to mass loss test. After immersion for 120 h, the samples were taken out from SBF and then blown dry with air at room temperature. The degraded surfaces were observed by SEM. Before the SEM observations, the samples were coated with gold by using a sputter coater (Leica EM SCD005, Leica Microsystems GmbH, Wetzlar, Germany).

### 3.3. Statistical Analysis

The experimental data of mechanical and degradation properties were expressed as mean ± standard deviation. Statistical analysis was performed to assess the difference by the analysis of variance. The difference was considered to be significant when *p* < 0.05.

## 4. Conclusions

The microstructure, degradation properties and hardness of the AZ61 magnesium alloys with different Y contents (0, 1, 2, 3, 4 wt %) prepared by laser rapid melting were investigated. Adding Y to AZ61 magnesium alloy could lead to the formation of Al_2_Y phase and reduce the amount of Mg_17_Al_12_ phase. The degradation resistance of the AZ61 magnesium alloy was enhanced with Y addition. The AZ61 magnesium alloy with 2 wt % Y exhibited an optimal degradation resistance. Furthermore, the hardness increased as Y contents increased from 0 wt % to 2 wt %, and then decreased when Y contents decreased further. In conclusion, laser rapid melting AZ61 magnesium alloy with 2 wt % Y exhibit prospects for future bone implants.
